# Over-expression of miR-146b and its regulatory role in intestinal epithelial cell viability, proliferation, and apoptosis in piglets

**DOI:** 10.1186/s13062-017-0199-9

**Published:** 2017-11-25

**Authors:** Xin Tao, Shujie Liu, Xiaoming Men, Ziwei Xu

**Affiliations:** Institute of Animal Husbandry and Veterinary Science, Zhejiang Academy of Agricultural Sciences, Hangzhou Zhejiang, 310021 People’s Republic of China

**Keywords:** MiR-146b, IPEC-J2, Proliferation, Apoptosis, TLR4

## Abstract

**Background:**

Weaning stress affects the small intestine of piglets. MiR-146b is differentially expressed in suckling and weaned piglets. In this study, we evaluated the effects of miR-146b on cell viability, proliferation, and apoptosis in IPEC-J2 cells.

**Results:**

Transfection with miR-146b mimics successfully increased miR-146b levels by 1000× (*P* < 0.001). The over-expression of miR-146b significantly promoted the apoptosis (*P* < 0.01) of IPEC-J2 cells, with no significant effects on cell viability or proliferation. MiR-146b suppressed the luciferase activity of the miR-TLR4-wt by 57% compared with the negative control, while mutation of the miR-146b binding site significantly blocked the suppressive effect (*P* < 0.05). Western blot results showed that TLR4 levels decreased in IPEC-J2 cells transfected with miR-146b mimics (*P* < 0.05).

**Conclusions:**

The over-expression of miR-146b promotes IPEC-J2 cell apoptosis. TLR4 is a direct target of miR-146b in IPEC-J2 cells.

**Reviewers:**

This article was reviewed by Eugene Berezikov and Jan B Hoek.

## Background

The weaning process, one of the most stressful events in the life of pigs, contributes to intestinal and immune system dysfunctions that negatively affect pig health, growth, and feed intake, particularly during the first week post-weaning [1]. Weaning activates stress signaling pathways that mediate intestinal dysfunction, impairs development of the intestinal mucosal barrier, enhances intestinal permeability, and leads to harmful inflammatory reactions [2–4].

MiRNAs are single-stranded non-coding small RNA molecules (~22 nucleotides), which regulate stress signaling pathways [5], intestinal epithelial tight junction, and intestinal permeability [6], control intestinal epithelial differentiation, architecture, and barrier function [7], and determine intestinal epithelial cell fate [8]. MiRNAs are involved in the regulation of immune responses and inflammatory diseases [9], including gut mucosal immunity [10] and inflammatory bowel diseases [11]. Using small RNA high-throughput sequencing technology, we have demonstrated that weaning results in a large number of differentially expressed miRNAs in piglets within the first week post-weaning [12]. MiR-146b, which is highly expressed, may play an important role in weaning-induced intestinal injuries in piglets. IPEC-J2 cells are porcine intestinal columnar epithelial cells that originated from neonatal piglet mid-jejunum and present similar characteristics to the intestinal epithelium [13]. To understand the role of miRNAs in the small intestine of weaned piglets, we investigated the expression and function of miR-146b in IPEC-J2 cells.

## Methods

### Cell culture

IPEC-J2 cells were cultured at 37 °C in a humidified incubator with 5% CO_2_ in 25 cm^2^ cell culture flasks (Corning, New York, NY, USA). Cells were grown in DMEM/F12 medium (Gibco, Carlsbad, NM, USA) supplemented with 10% heat-inactivated fetal bovine serum (Gibco, Carlsbad, NM, USA) supplemented with 1% penicillin-streptomycin (Sigma, St. Louis, MO, USA) and 1% glutamine (Amersco, Solon, Tucson, AZ, USA). The culture medium was changed every other day.

### Cell transfection

Cells were seeded into cell culture plates (Corning, New York, NY, USA) at 1 × 10^5^ cells/ml for 24 h prior to transfection. The miRNA mimics for miR-146b and negative control (sense sequence: UUCUCCGAACGUGUCACGUTT; antisense sequence: ACGUGACACGUUCGGAGAATT) were purchased from RiboBio Co. (Guangzhou, China). Cells were grown to 50% confluence and transiently transfected with 50 nM miR-146b mimics or 50 nM negative control (NC) mimics or blank control (no treatment) using Lipofectamine 3000 in Opti-MEM (Invitrogen, Carlsbad, CA, USA) according to the manufacturer’s instruction. Cell samples were collected 48 h after the transfection.

### Cell viability and proliferation assay

#### CCK-8 assay

We performed the CCK-8 assay to assess the effects of miR-146b on IPEC-J2 cell viability. First, cells were transfected and cultured in 12-well culture plates. At the end of transfection, isolated cells were seeded onto 96-well culture plates at 600 cells per well in DMEM/F12 medium. CCK-8 (10 μL; Dojindo, Kumamoto, Japan) was added to each well and incubated for 1.5 h at 37 °C in 5% CO_2_. We estimated viable cell number by measuring optical density at 450 nm using a Multiskan MK3 microplate reader (Thermo, Waltham, MA, USA). The amount of the formazan dye, generated by the viability of cellular dehydrogenises, is directly proportional to the number of living cells.

#### CFSE assay

We measured cell proliferation by the carboxyfluorescein diacetate succinimidyl ester (CFSE) method [14, 15]. CFSE (Invitrogen, Carlsbad, USA) was dissolved in dimethyl sulfoxide (DMSO) to make a 5 mM stock solution. And CFSE solution was added to the cells (2 × 10^7^) at a final concentration of 10 μM. The cells were subsequently stained at 37 °C for 30 min, diluted with 5 volumes of ice-cold culture medium, centrifuged, washed by re-suspending in fresh medium, and plated at 5 × 10^5^ cells/well in a 24-well plate. The following day, CFSE-labeled cells were transfected as described above. After transfection for 48 h, cultured cells were harvested, washed twice with PBS, and analyzed using a FACS Calibur flow cytometer (BD Biosciences, San Jose, USA). Data analysis was performed using ModFit software (Verity Software House, Toshan, USA).

### Apoptosis detection

Cells were transfected and cultured for 48 h. Apoptosis was detected using the Annexin V-FITC apoptosis detection kit (Invitrogen, Carlsbad, USA). Briefly, cells were washed in ice-cold PBS and re-suspended in 1× binding buffer in the presence of Annexin FITC-V and propidium iodide and incubated at room temperature for 15 min in the dark. The stained cells were analyzed by BD flow cytometry in the presence of FACS.

### Construction of 3′-UTR reporter plasmids and luciferase assay

The miR-146b target site was predicted using the miRanda algorithm (http://www.microrna.org/). The 3′UTR of the TLR4 gene containing miR-146b binding sites were amplified by PCR from genomic DNA of IPEC-J2 cells (TLR4-F: GATCTCGAGGGAGGGAAAACTCCCAACGTGTC, TLR4-R: AATGCGGCCGCTGACATCAAGTGACAAAGTGACAGTG) and cloned into the XhoI and NotI sites downstream of the luciferase reporter gene of the pmiR-RB-ReportTM vector (RiboBio, Guangzhou, China), which was labeled pmiR-TLR4 -wt. Mutations in the predicted miR-146b binding sites were performed using by PCR-based site-directed mutagenesis with miR-TLR4-wt as a template (pmiR-TLR4-mut). All constructs were verified by DNA sequencing. IPEC-J2 cells were co-transfected in 24-well plates with either miR-146b mimics or NC mimics and miR-TLR4-wt or pmiR-TLR4-mut by Lipofectamine 3000. After 48 h, luciferase activity was measured using a dual luciferase assay system (Promega, Heidelberg, Germany). The firefly luciferase activity of each sample was normalized to Renilla luciferase activity.

### RT-qPCR

Total RNA was extracted using E.Z.N.A. HP Total RNA kit (Omega Bio-Tek, Norcross, USA), and total RNA concentration was determined in a NanoDrop1000 spectrophotometer (Thermo Fisher Scientific, Wilmington, USA). ReverTra Ace reverse transcriptase (Toyobo, Osaka, Japan) and miRNA-specific were used to synthesize cDNA. The stem-loop RT-qPCR method was performed, stem-loop RT primers for miR-146b were designed [12], and U6 snRNA was used as an internal control [16]. RT-qPCR was performed using SYBR Green Real-time PCR Master Mix (Toyobo, Osaka, Japan) and ABI StepOne Plus real-time PCR system (Applied Biosystems, Foster City, USA). All reactions were carried out in triplicate. Relative quantification was calculated using 2^-ΔΔCt^.

### Western blot

Protein was isolated from IPEC-J2 cells, which were transiently transfected and cultured for 48 h, using a total protein extraction kit. Protein concentration was determined using a BCA kit (Sangon Biotech, Shanghai, China). A total of 100 μg protein per lane was subjected to 12.5% SDS-PAGE, electroblotted onto an electrochemiluminescent nitrocellulose membrane, and analyzed by Western blotting. TLR4 protein levels were quantified using a mouse anti-TLR4 monoclonal antibody (Abcam, Cambridge, UK). GAPDH (internal control) was quantified using mouse anti-GAPDH monoclonal antibody (Abcam). Band signals were acquired in the linear range of the scanner and analyzed using QUANTITY ONE software (Bio-Rad, Hercules, USA).

### Statistical analysis

We analyzed the data by one-way ANOVA using SPSS software (SPSS, Chicago, USA). Comparisons between groups were carried out by LSD (for data with equal variances) or Dunnett’s T3 (for data with unequal variances). Results are presented as mean ± SE. *P* < 0.05 was considered statistically significant.

## Results

### Over-expression of miR-146b in IPEC-J2 cells

**Fig. 1:**
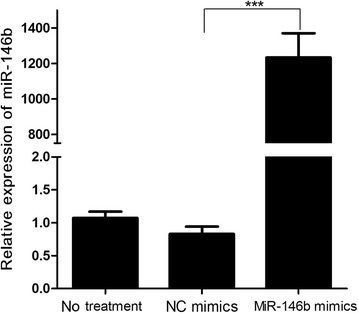
MiR-146b expression in IPEC-J2 cells. IPEC-J2 cells were transfected with miR-146b mimics or negative control (NC) mimics at a final concentration of 50 nM for 48 h. Relative expression of miR-146b was quantified by real-time PCR. Data were obtained from three independent experiments performed in triplicate. ***P < 0.001

To investigate the expression and functions of miR-146b in IPEC-J2 cells, we carried out a gain-of-function experiment by transfecting miR-146b mimics or NC mimics into IPEC-J2 cells. The RT-qPCR results showed that the expression of miR-146b was enhanced (more than 1000×) in miR-146b mimic-transfected cells than in NC mimic-transfected cells (*P* < 0.001, Fig. 1). Therefore, miR-146b was successfully over-expressed in IPEC-J2 cells by transfection.

### Effect of miR-146b on IPEC-J2 cell viability, proliferation, and apoptosis

**Fig. 2:**
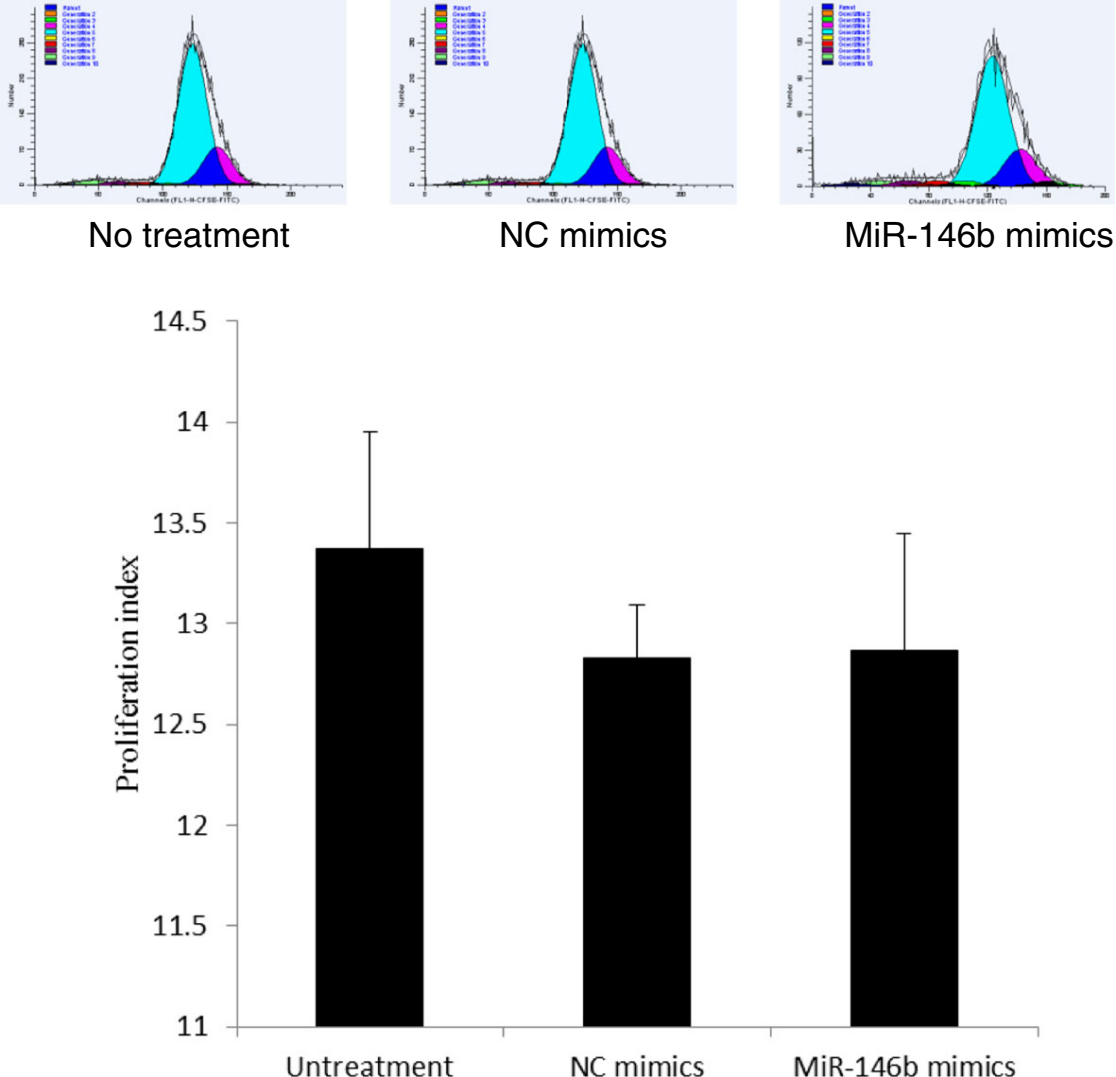
Effects of miR-146b on IPEC-J2 cell proliferation. IPEC-J2 cells were transfected with miR-146b mimics or negative control (NC) mimics at a final concentration of 50 nM for 48 h. The cells were treated using a carboxyfluorescein diacetate succinimidyl ester (CFSE) proliferation assay. The percentages of CFSE-labeled cells were analyzed using FACS Calibur flow cytometer. Data were obtained from three independent experiments performed in triplicate

To study the effect of miR-146b on IPEC-J2 cell proliferation, we performed a cell CFSE proliferation assay. The results of FACS analysis showed that the miR-146b mimics had no significant effects on the proliferation of IPEC-J2 cells compared with the NC mimics (*P* > 0.05, Fig. 2).

**Fig. 3:**
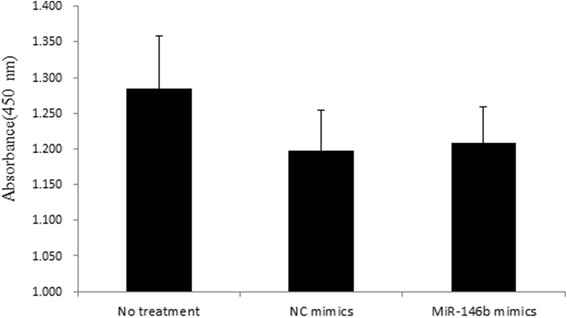
Effects of miR-146b on IPEC-J2 cell vitality. IPEC-J2 cells were transfected with miR-146b mimics or negative control (NC) mimics at a final concentration of 50 nM for 48 h. The cells were analyzed for vitality using a CCK-8 assay. The viable cell number was determined by measuring optical density. Data were obtained from three indepenent experiments performed in triplicate

To assess the effect of miR-146b on IPEC-J2 cell viability, we performed a CCK-8 assay. The results showed that over-expression of miR-146b had no significant effects on cell activity compared with NC mimics (*P* > 0.05, Fig. 3).

**Fig. 4:**
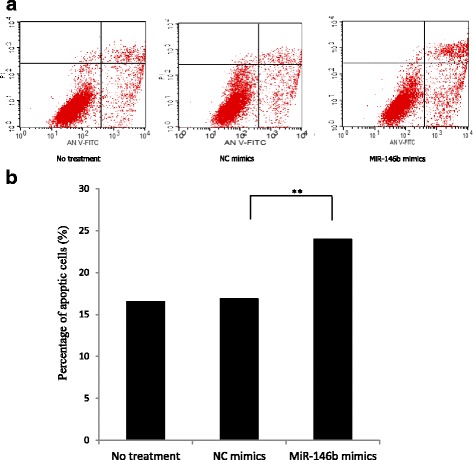
Effects of miR-146b on IPEC-J2 cell apoptosis. IPEC-J2 cells were transfected with miR-146b mimics or negative control (NC) mimics at a final concentration of 50 nM for 48 h. The cells were analyzed for apoptosis by staining with FITC-Annexin V. The percentages of Annexin V-labeled cells were analyzed using FACS Calibur flow cytometer. Data were obtained from three independent experiments performed in triplicate. Values are expressed as mean ± SE, ** *P* < 0.01

Finally, to determine the effect of miR-146b on IPEC-J2 cell survival, we stained IPEC-J2 cells using the Annexin V-FITC apoptosis detection kit. The results revealed that the over-expression of miR-146b increased apoptosis by 41.98% compared with NC mimics (*P* < 0.01, Fig. 4).

### TLR4 3’UTR contains a conserved binding site for miR-146b

MiRanda algorithm was used to scan targets for miR-146b [17]. The thresholds for candidate target sites were S > 90 and ΔG < −17 kcal/mol. S represents the single-residue-pair match score over the alignment trace, and ΔG represents the free energy of duplex formation from a completely dissociated state. The calculations were performed using the Vienna Package [18]. Gene 3’UTR sequences from swine genomic data were obtained from the Ensembl database. We obtained 45 possible targets for miR-146b (Table 1). Among the possible targets, TLR4 attracted our interest because of its role in cytoprotection during stressful conditions [19–21].

**Table 1:** Target genes of miR-146b

miRNA	cDNA Seq	Name	Gene Name	Tot Score
ssc-miR-146b	ENSSSCT00000000314	F1SR53_PIG	similar to integrin, alpha 5 (fibronectin receptor, alpha polypeptide)	152
ssc-miR-146b	ENSSSCT00000000399	F1SPH5_PIG	ribosomal protein S26	143
ssc-miR-146b	ENSSSCT00000000438	F1SLA0_PIG	similar to ATP synthase subunit beta, mitochondrial	141
ssc-miR-146b	ENSSSCT00000000455	F1SL74_PIG	similar to Serine hydroxymethyltransferase 2 (mitochondrial)	149
ssc-miR-146b	ENSSSCT00000000530	LYSC2_PIG	lysozyme (renal amyloidosis)	144
ssc-miR-146b	ENSSSCT00000000748	F1SLT4_PIG	CD4 molecule	155
ssc-miR-146b	ENSSSCT00000001516	PO5F1_PIG	POU class 5 homeobox 1	155
ssc-miR-146b	ENSSSCT00000001590	A5A8X7_PIG	pre-B-cell leukemia homeobox 2 pseudogene 1	145
ssc-miR-146b	ENSSSCT00000001619	F1RQ22_PIG	MHC class II, DO beta	155
ssc-miR-146b	ENSSSCT00000001841	MEA1_PIG	male-enhanced antigen 1	140
ssc-miR-146b	ENSSSCT00000001954	B6E279_PIG	interleukin 17A	142
ssc-miR-146b	ENSSSCT00000001960	Q29096_PIG	proteasome (prosome, macropain) subunit, alpha type, 4	147
ssc-miR-146b	ENSSSCT00000002636	F1S2T2_PIG	similar to YLP motif containing 1	140
ssc-miR-146b	ENSSSCT00000002650	FOS	v-fos FBJ murine osteosarcoma viral oncogene homolog	143
ssc-miR-146b	ENSSSCT00000004461	D0G0C1_PIG	1-acylglycerol-3-phosphate O-acyltransferase 4 (lysophosphatidic acid acyltransferase, delta)	154
ssc-miR-146b	ENSSSCT00000005288	F1SS26_PIG	thrombospondin 1	142
ssc-miR-146b	ENSSSCT00000005528	ARF6_PIG	ADP-ribosylation factor 6	154
ssc-miR-146b	ENSSSCT00000005554	F1SFE6_PIG	similar to proteasome 26S ATPase subunit 6	171
ssc-miR-146b	ENSSSCT00000006051	F1SMH9_PIG	toll-like receptor 4	161
ssc-miR-146b	ENSSSCT00000006601	F1S1K0_PIG	similar to Double-strand-break repair protein rad21 homolog (hHR21) (Nuclear matrix protein 1) (NXP-1) (SCC1 homolog)	147
ssc-miR-146b	ENSSSCT00000006734	CAH3_PIG	carbonic anhydrase III, muscle specific	144
ssc-miR-146b	ENSSSCT00000006859	F1RSG9_PIG	similar to Lysophospholipase I	149
ssc-miR-146b	ENSSSCT00000006890	F1RPW3_PIG	selectin P (granule membrane protein 140 kDa, antigen CD62)	142
ssc-miR-146b	ENSSSCT00000007016	F1RJ76_PIG	C-reactive protein	145
ssc-miR-146b	ENSSSCT00000007067	A0ZQ05_PIG	CD1d molecule	149
ssc-miR-146b	ENSSSCT00000007082	F1RHJ5_PIG	similar to Insulin receptor-related protein precursor (IRR) (IR-related receptor)	150
ssc-miR-146b	ENSSSCT00000007490	F1S5Y9_PIG	similar to TAF13 RNA polymerase II, TATA box binding protein (TBP)-associated factor, 18 kDa	144
ssc-miR-146b	ENSSSCT00000008224	A5GFP6_PIG	prostate transmembrane protein, androgen induced 1	147
ssc-miR-146b	ENSSSCT00000008233	F1RJR6_PIG	syntaxin 16	157
ssc-miR-146b	ENSSSCT00000010570	A7XNM3_PIG	stanniocalcin 1	142
ssc-miR-146b	ENSSSCT00000010801	F1RKG3_PIG	similar to replication factor C 5	146
ssc-miR-146b	ENSSSCT00000010839	ALDH2_PIG	aldehyde dehydrogenase 2 family (mitochondrial)	149
ssc-miR-146b	ENSSSCT00000010959	F1RFD3_PIG	similar to Splicing factor 3 subunit 1 (Spliceosome-associated protein 114) (SAP 114) (SF3a120)	141
ssc-miR-146b	ENSSSCT00000011263	F1SU77_PIG	similar to Group XIIB secretory phospholipase A2-like protein precursor (Group XIII secretory phospholipase A2-like protein) (GXIII sPLA2-like) (sPLA2-GXIIB) (GXIIB)	155
ssc-miR-146b	ENSSSCT00000011288	VINC_PIG	vinculin	
ssc-miR-146b	ENSSSCT00000011384	MSMB_PIG	microseminoprotein, beta-	141
ssc-miR-146b	ENSSSCT00000011457	MARCH5	similar to E3 ubiquitin-protein ligase MARCH5 (Membrane-associated RING finger protein 5) (Membrane-associated RING-CH protein V) (MARCH-V) (RING finger protein 153)	156
ssc-miR-146b	ENSSSCT00000011467	F1SC80_PIG	retinol binding protein 4, plasma	149
ssc-miR-146b	ENSSSCT00000011523	F1S8W9_PIG	similar to Probable oxidoreductase C10orf33	142
ssc-miR-146b	ENSSSCT00000011546	Q6RWA7_PIG	stearoyl-CoA desaturase	141
ssc-miR-146b	ENSSSCT00000011756	F1SDM5_PIG	similar to DEAD/H (Asp-Glu-Ala-Asp/His) box polypeptide 32	144
ssc-miR-146b	ENSSSCT00000012349	F1SRG7_PIG	vasoactive intestinal peptide receptor	142
ssc-miR-146b	ENSSSCT00000013040	F1SP93_PIG	ATPase, H+ transporting, lysosomal 70 kDa, V1 subunit A	140
ssc-miR-146b	ENSSSCT00000013586	F1RPJ7_PIG	similar to uracil phosphoribosyltransferase (FUR1) homolog	141
ssc-miR-146b	ENSSSCT00000017203	F1RZN7_PIG	kallikrein	144

### TLR4 is a target for miR-146b

**Fig. 5:**
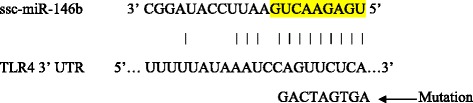
Ssc-miR-146b binding site in corresponding 3’UTR sequence of swine predicted target gene TLR4 (seed sequence highlighted in yellow). The position of this binding site is located from 559 to 579 bp in the 3’UTR sequence (Genbank accession No: AB188301). The potential target gene was identified by luciferase assay

**Fig. 6:**
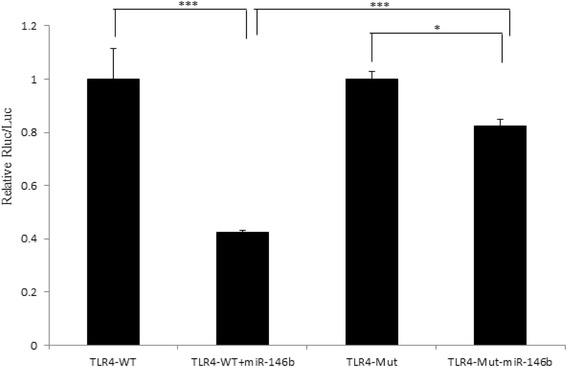
Luciferase activity of various reporters in the presence and absence of miR-146b mimics. The ratio of reporter (Renilla lucifearse, Rluc) to control plasmid (Firefly luciferase, Luc) in relative luminescence units was normalized to the control value from the control transfection (*n* = 3). ***P < 0.001; *P < 0.05

Bioinformatics analysis revealed that there was one putative miR-146b binding site (Fig. 5). To verify whether the predicted miR-146b-binding site in the 3’UTR of TLR4 mRNA was responsible for its regulation, we cloned the 3’UTR region of TLR4 into pmiR- REPORT luciferase reporter vector and co-transfected this vector together with miR-146b mimics or NC mimics into IPEC-J2 cells. The results showed that the luciferase activity decreased by 57% in cells transfected with miR-146b mimics compared to cells transfected with NC mimics (*P* < 0.001; Fig. 6). Mutation of the predicted miR-146b-binding sites blocked the miR-146b-induced suppression of luciferase activity (*P* < 0.05), and was significantly increased compared to the wild type of miR-146b (P < 0.001). These results revealed that miR-146b may regulate the expression of TLR4 by targeting its 3’UTR region at the post-transcriptional level; however, there are other miR-146b target sites in TLR4 except the mutation site of this study.

### MiR-146b inhibits the expression of TLR4 protein

**Fig. 7:**
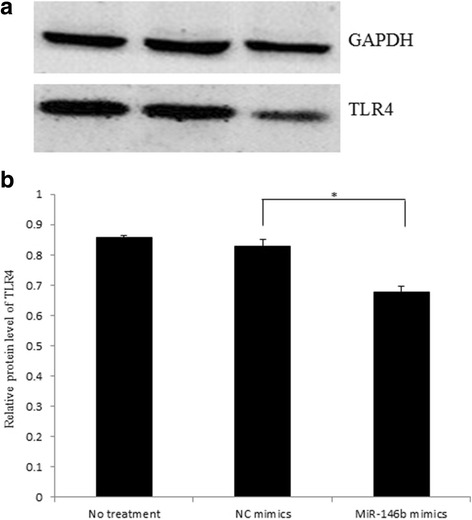
Expression of TLR4 protein in IPEC-J2 by transfection with miR-146b mimics. **a** Down-regulation of TLR4 protein in IPEC-J2 cells by miR-146b mimics was detected by Western blot. **b** Intensities of each protein band were quantified by Quantity Software. Data were obtained from three independent experiments performed in triplicate. *P < 0.05

Western blot for TLR4 protein showed that the expression level of TLR4 was separately reduced by 18.37% and 21.04% in miR-146b mimic-transfected cells compared to NC mimic-transfected and non-transfected cells (*P* < 0.05; Fig. 7a and b). There were no significant differences in the levels of TLR4 protein between the NC mimic-transfected cells and non-transfected cells (*P* > 0.05).

## Discussion

Weaning stress induces injury in the intestinal mucosal barrier, leading to increased permeability, poor health, and impaired growth of piglets [1]. Intestinal epithelial cells (IECs) are important components of the intestinal mucosal barrier and are crucial mediators of intestinal homeostasis that enable the colonization by commensal bacteria [22]. In this study, we demonstrated the expression of miR-146b in IECs of piglets. Our previous findings revealed that the expression of miR-146b was the most up-regulated in the small intestine of weaned piglets [12]. These results showed that expression of miR-146b in IECs of weaned piglets may play a critical role in stress-induced intestinal injury.

MiR-146a and miR-146b are members of the miR-146 family located on chromosomes 16 and 14 of pigs, respectively. The sequences of these mature miRNAs are consistent with those of humans, except that the 5’end of ssc-miR-146b is lack of a base “U” [23]. Only two nucleotides are different between miR-146a and miR-146b; therefore, they have similar target genes. In recent years, studies have revealed that miR-146 regulates the inflammatory response of human cells, including retinal pigment epithelial cells, adipocytes, and monocytes [24–26]. MiR-146a/b is expressed in the duodenal epithelium of Vibrio cholera-infected patients at the acute stage of cholera and in the small intestine of weaned piglets [12, 27]. However, the role of miR-146b in the IECs of piglets has not been elucidated.

Our findings revealed that miR-146b affected the apoptosis of IPEC-J2 cells. Transfection with miR-146b mimics significantly promoted the apoptosis of IPEC-J2 cells, with no significant effects on cell viability or proliferation. MiR-146b is actively induced by TGF-β-induced IEC-6 differentiation [28]. In visceral preadipocytes, miR-146b inhibits cell proliferation and promotes differentiation [29]. Similarly, miR-146b inhibits the proliferation and promotes the differentiation of human visceral preadipocytes and enhances the proliferation of vascular smooth muscle cells and esophageal cancer cells [30, 31]. This may be closely related to different types and functions of cells. Our findings demonstrated the role of miR-146b in IEC of piglets, which is mainly related to target genes regulated by miR-146b. Additionally, in this study we found the overexpression of miR-146b has no effect on cell proliferation but increases cell apoptosis. This is largely influenced by the incubation conditions including the concentrations of miR-146b transfection and the subsequent culture time of IPEC-J2 cells. Consistent with our results, overexpressing miR-146a and/or miR-146b was found to increase the apoptosis of human dendritic cells, despite no results of cell proliferation [26]. Further complementary work is needed to determine the accurate functions of different concentrations of miR-146b transfection and preferable culture time of IPEC-J2 cells.

TRAF6 and IRAK1, which are two key adapter molecules in the TLR4 signaling pathway, have been identified to be targets of miR-146 [32]. MiR-146 plays a vital role in immune responses and anti-inflammation by primarily targeting TRAF6 and/or IRAK1 of the TLR4/NF-κβ signaling pathway [33–36]. Furthermore, miR-146a prevents ischemia/reperfusion of small intestine injury by downregulating IRAK1 expression [37], and miR-146a/b prevent liver graft injury in small-for-size liver graft injury in rats by inactivating IRAK1 and TRAF6 [38]. Although TLR4 has been demonstrated to be a direct target of miR-146b in human monocytes [39], the function of miR-146 by directly targeting TLR4 is relatively rare reported. In this study, we demonstrated that TLR4 is a direct target of miR-146b in porcine IECs. Specifically, miR-146b decreased TLR4 protein expression. Therefore, the anti-inflammatory and immune-regulatory effects of miR-146b might result from not only targeting IRAK1 and TRAF6, but also targeting TLR4 in the small intestine of weaned piglets. In addition, the apoptosis induced by miR-146b mimics might be related to lower TLR4 protein expression in view of its cytoprotective effects [19–21]. However, this study mainly focused on the expression and effects of miR-146b on cell viability, proliferation, and apoptosis in normal IEC cells, further studies are needed to verify the underling mechanism in IEC cells under certain stress conditions.

## Conclusions

Over-expression of miR-146b promotes IPEC-J2 cell apoptosis with no significant effects on IPEC-J2 cell viability and proliferation. TLR4 is a direct target of miR-146b in IPEC-J2 cells. Transfection with miR-146b mimics decreased the expression level of TLR4 protein.

## Reviewers’ comments

### Reviewer’s report 1: Eugene Berezikov, University of Groningen, Netherlands

Reviewer’s comments: The process of weaning, or transition from sucking milk to an adult diet, is a stressful event that can negatively affect animal well-being. In this work, the authors study molecular mechanisms underlying this transition in piglets. In a previous work, the authors investigated changes in microRNA expression during weaning, and identified miR-146b as highly expressed. Here, they used porcine intestinal epithelial cell line IPEC-J2 to functionally characterize this miRNA. The authors show that overexpression of mir-146b does not affect cell proliferation or vitality but increases levels of apoptosis. Furthermore, the authors demonstrate that TLR4 is a direct target of miR-146b. Although mir-146b targeting of TLR4 was previously shown in human monocytes, this work expands it porcine cells.

Apoptosis and proliferation are usually connected but in this work the overexpression of mir-146b has no effect on proliferation but increases apoptosis. This needs to be at the very minimum discussed.

Author’s response: *Following the reviewer’s suggestion, we have discussed the result that the overexpression of mir-146b has no effect on proliferation but increases apoptosis.*

*Corresponding changes in the manuscript: p.13.*

1. Legends for Figs. 2, 3 and 5 would benefit from a more detailed explanation. ‘Untreatment’ should be ‘non-treated’.

Author’s response: *Following the reviewer’s and the other reviewer’s suggestion, we have made the corresponding supplement and modification.*

*Corresponding changes in the manuscript: p.32.*

2. Lines 149 and 176, mention by how much there is increase/decrease of the effect.

Author’s response: *Following the reviewer’s suggestion, the percentage values of increase/decrease of the effect has been calculated and added.*

*Corresponding changes in the manuscript: p.9, 11.*

3. Table 1. Would be useful to include target prediction scores.

Author’s response: *Following the reviewer’s suggestion, target prediction scores have been added.*

4. Fig. 5. Would be useful to indicate the relative position of the target site in the 3’UTR.

Author’s response: *Following the reviewer’s suggestion, the relative position of the target site in the 3’UTR has been added.*

### Reviewer’s report 2: Jan B Hoek, PhD

Reviewer’s comments: I find the article to be of modest interest largely due to its limited scope. It relies on a very high level of overexpression of the miRNA mimic, which is not being commented on. Given this high level of overexpression the impact on TLR4 expression is modest (by the 3′-UTR luciferase assay) and more so at the level of the endogenous TLR4 protein. Some degree of suppression is evident even with the mutant 3′-UTR-TLR4, suggesting that other interactions may play a role, which are not further explored. There is no information on dose dependency or time course of the treatment with the miR-146b mimic. Also, the critically important validation of a change in TLR4 expression in the piglet epithelium upon weaning at a time point when miR-146b is elevated is lacking. How these findings relate to the physiological changes upon weaning in the piglet is therefore unclear. Thus the findings reported here are limited to a test in a transfected cell system that suggests some targeting of TLR4 by this microRNA, without good evidence of physiological relevance.

This paper builds on earlier studies by this group, notably the 2013 paper in PLoS One (ref 12) where the authors reported changes in the microRNA profile of intestinal epithelium in piglets upon weaning. In those studies, among a large number of microRNAs that were up- or down-regulated within a period of 4–7 days after weaning, miR-146b stood out as the most highly upregulated microRNA species, with a relative increase (compared to non-weaned suckling age-comparable counterparts) of 5–10 fold. The weaning-induced changes in microRNA profile were associated with considerable changes in the innate immune response well as increased intestinal permeability and other physiological stress responses. In the present study the authors aim to identify potential targets of miR-146b by evaluating candidate targets identified by the miRanda algorithm focusing on potential binding sites in the 3′-UTR of candidate genes. Out of the 45 or so candidate targets identified by the algorithm, they focus their attention on TLR4 and carry out experiments in the IPEC-J2 cell line (derived from swine intestinal epithelium) by transfection with a miR-146b mimic and using a luciferase reporter construct to assess interaction with the 3′-UTR of TLR-4. The data indicate that transfection with the MiR-146b mimic, but not with a non-matching microRNA mimic modestly increased cellular apoptosis, but had no significant effect on cell proliferation and cell viability, compared to NC mimic transfected cells. MiR-146b transfection also significantly suppressed luciferase expression in cells expressing the WT 3′-UTR, whereas a mutated 3′-UTR TLR4 construct was considerably less affected (although still significant, suggesting other interactions remained). Protein levels of endogenous TLR4 were also suppressed in cells transfected with the miR-146b mimic. The authors conclude that miR-146b is a potential modulator of the innate immune response not only through its effects on TRAF6 and IRAK1 (as had been demonstrated earlier in other cell types), but also through direct suppression of TLR4. The paper is technically sound and makes effective use of the IPEC-J2 swine epithelial cell line, which is a non-transformed non-tumorigenic cell line that is widely used as an effective model of the pig epithelium in vivo. Also, the conclusion that TLR4 is a potential direct target of miR-146b under the conditions used here is supported by the data. This is not necessarily surprising since a previous study (ref 39) had reported that TLR4 is a direct target of miR-146b in human monocytes. Apart from the question of novelty, however, there are several issues that make the paper less compelling.

1. The paper relies on a very pronounced overexpression of miR-146b (1000-fold over basal). This compares to a 5–10-fold increase in expression in the piglet model in the intestinal epithelium at 4–7 days after weaning. Given this high degree of overexpression, the suppression of TLR4 expression would seem to be limited (about 60% in the luciferase assay, less than 15% at the protein level). The authors do not provide information on the dose dependency or the time course of the effect of miR-146b mimic transfection. Presumably all these transfection experiments were carried out at 48 h after transfection with a single dose of the mimic (although detailed information on treatment and incubation conditions is limited, see below). Did the authors test the effects of miR-146b at lower expression levels and/or at other time points? In particular, the impact on TLR4 protein levels may be evident only after longer incubation times.

Author’s response: *We very much appreciate the overall comments of the reviewer. First, the miR-146b mimic concentration of 50 nM is recommended by the manufacturer (RiboBio CO., Guangzhou, China). Secondly, we initially evaluated the transfection efficiency of miR-146b mimics mainly by real-time PCR assay. Three concentrations of miR-146b mimics were set at 10 nM, 50 nM and 100 nM. The real-time PCR results showed the overexpression levels of miR-146b at the two doses of 50 nM and 100 nM were very significantly higher than 10 nM, but there is a smaller difference between 50 nM and 100 nM. Thirdly, we read plenty of studies which all used other miRNA mimic products from this manufacturer (RiboBio CO., Guangzhou, China) before the cell transfection experiments. These studies also reported only the dose of 50 nM such as miR-133b in human oocyte (PLoS ONE, 2014, 9(6):e100751), miR-7 in NSCLC cell line (International Journal of Biological Sciences, 2011, 7(6):805), miR-30a in type II alveolar epithelial cells (Journal of Cellular and Molecular Medicine, 2014, 18(12):2404), and so on. Therefore, we finally selected the dose of 50 nM in all the assays.*

2. A question with all such cell line experiments is to what extent the cell lines mimic the response capacity of the piglet epithelium in vivo, and more particularly the stress state of the epithelial cells following weaning. Do the authors have any information on the gene expression profile of IPEC-J2 cells compared to intact piglet epithelium? In particular, in view of the difference in fold expression of miR-146b in the cell system compared to piglet epithelium after weaning, the question is relevant what the basal level of TLR4 is in the cell line relative to the in vivo system. Did the authors attempt to quantify relative TLR4 expression? Also, did the authors test how TLR4 expression in the pig epithelium responds to weaning over the 4 day or 1 week period used in their 2013 publication?

Author’s response: *we are very sorry that we haven’t studied on the gene expression profile of IPEC-J2 cells compared to intact piglet epithelium, and we also haven’t searched any corresponding references by now. However, we did detect the mRNA expression of TLRs in the small intestinal epithelium of piglets over the first week post-weaning, and the results had been published elsewhere (Anaerobe, 2015, 32:51–56). Because the IPEC-J2 cells used in the present study didn’t be studied in stressful conditions, the gene expression profile of IPEC-J2 cells couldn’t be compared to the previous study* in vivo*. Furthermore, the main purpose of this study is to understand if miR-146b can be over-expression and play a regulatory role in intestinal epithelial cell functions, and verify if TLR4 is a direct target of miR-146b in IPEC-J2 cells. Certainly, we have the same opinion with the reviewer, the gene expression profile of IPEC-J2 cells should be compared to the corresponding results of small intestinal epithelium in pigs. This is also one of our research objectives in the future. In fact, we have already begun to study on miR-146b in LPS stimulated IPEC-J2 cells.*

3. It is not quite clear what the underlying hypothesis is regarding the contribution of miR-146b to the stress response in the intestinal epithelium in vivo. Presumably the concept is that TLR4 activation is a contributing factor to the intestinal epithelial damage and that upregulation of miR-146b could counteract that condition. However, the authors also suggest that TLR4 may have a cytoprotective effect. Did the authors make an attempt to induce stress in the IPEC-J2 cells in vitro, e.g. by treatment with LPS, to assess a possible protective effect of miR-146b overexpression? This may also provide a better test of the dose dependency of any suppressive action and enable the authors to determine the contribution of TLR4 suppression as compared to the effects of suppression of other candidate downstream mediators, such as TRAF6 and IRAK1.

Author’s response: *Thanks for the kind suggestion. In fact, we have already begun to study on miR-146b in LPS stimulated IPEC-J2 cells. The conditions of LPS stimulating IPEC-J2 cells including the length of time and the dose have been definite. The transfection experiments of miR-146b mimics are being done in LPS stimulated IPEC-J2 cells. The main goal of the future experiments will focus on the functions of miR-146b and the target genes (TLR4, TRAF6 and IRAK1) in the intestinal epithelial cells under stressful conditions.*

4. The authors make a point of emphasizing the pro-apoptotic effect of miR-146b transfection. However, this effect appears to be relatively modest (an increase from 17% to 23% in Fig. 4) and there is no further analysis of the underlying mechanism. The authors suggest that the suppression of TLR4 may play a role, but do not provide further evidence to back this up. In fact, the relatively modest decrease in endogenous TLR4 protein level by overexpression of miR-146b (Fig. 7) does not support the argument that the TLR4 protein is a significant contributor to the apoptosis induced by miR-146b overexpression and the authors provide no further evidence that these events are causally related. Again, further time course or dose dependency data might have provided information to support (or not) the mutual dependency of these events. Presumably miR-146b has a multitude of other targets in the cell line that may have contributed to the increase in apoptosis.

Author’s response: *Thanks for the reviewer’s valuable comments. We found that we still have a few deficiencies in the current work. In future study, we will supply some assays about the time course and doses of miR-146b mimics to provide more evidence for our study. Furthermore, we have discussed an increase in apoptosis and their implication for the interpretation of the findings reported in the discussion part according to the reviewer’s suggestion. Corresponding changes in the manuscript: p.13.*

Minor comments: 1. The experimental detail provided regarding incubation conditions of the IPEC-J2 cells is rather minimal, e.g., the length of time of incubation after transfection should be included in the descriptions in the Methods. Also, the information on the sequence of the negative control mimic is lacking from the Methods section.

Author’s response: *Following the reviewer’s suggestion, the experimental details have been provided in the Methods including the length of time of incubation after transfection and the information on the sequence of the negative control mimic.*

*Corresponding changes in the manuscript: p.4.*

2. The authors detect an increase in apoptosis (by Annexin-V binding) by transfection with the miR-146b mimic compared to the NC mimic, but no significant difference in cell viability. However, the statistical error in the viability tests shown in Fig. 3 would seem to be large and it may be difficult to detect an increase in cell death that could be due to the modest increase in apoptosis. Also, the relatively high rate of apoptosis in the untreated cells is a reason for concern, suggesting that incubation conditions may be suboptimal. The authors should comment on these observations and their implication for the interpretation of the findings reported here.

Author’s response: *Following the reviewer’s suggestion, we have added the discussion on these observations and their implication for the interpretation of the findings reported in the discussion part.*

*Corresponding changes in the manuscript: p.13.*

3. The percentage change is cited at an unrealistic degree of precision, i.e. a decrease of 57.56% in the luciferase data is mentioned in the abstract and on p.10 when the error bar for the WT condition is >10%. This number should be given as 57%. 4. There are a few language issues. “Vitality” is used where “viability” would be the standard term. In the figures, the term “Untreatment” should be changed to “No treatment”. The sentence on p. 11/line 193–194 (..the 5’end of ssc-miR-146b is less than a base “U”…) is not clear and needs to be reformulated.

Author’s response: *Following the reviewer’s suggestion, we have modified the above issues.*

## Abbreviations

IECs: Intestinal epithelial cells

## References

[1] Campbell JM, Crenshaw JD, Polo J (2013). The biological stress of early weaned piglets. J Anim Sci Biotechnol.

[2] Moeser A, Vander Klok C, Ryan KA, Wooten JG, Little D, Cook VL (2007). Stress signaling pathways activated by weaning mediate intestinal dysfunction in the pig. Am J Physiol Gastrointest Liver Physiol.

[3] Lambert GP (2009). Stress-induced gastrointestinal barrier dysfunction and its inflammatory effects. J Anim Sci.

[4] Smith F, Clark JE, Overman BL, Tozel CC, Huang JH, Blikslager AT (2010). Early weaning stress impairs development of mucosal barrier function in the porcine intestine. Am J Physiol Gastrointest Liver Physiol.

[5] Mendell JT, Olson EN (2012). MicroRNAs in stress signaling and human disease. Cell.

[6] Ye D, Guo S, Al-Sadi R, Ma TY (2011). MicroRNA regulation of intestinal epithelial tight junction permeability. Gastroenterology.

[7] Mckenna LB, Schug J, Vourekas A, McKenna JB, Bramswig NC, Friedman JR (2010). MicroRNAs control intestinal epithelial differentiation, architecture, and barrier function. Gastroenterology.

[8] Dalmasso G, Nguyen HT, Yan Y, Laroui H, Srinivasan S, Sitaraman SV (2010). MicroRNAs determine human intestinal epithelial cell fate. Differentiation.

[9] Sonkoly E (2009). Pivarcsi a. microRNAs in inflammation. International Reviews of Immnology.

[10] Biton M, Levin A, Slyper M, Alkalay I, Horwitz E, Mor H (2011). Epithelial microRNAs regulate gut mucosal immunity via epithelium-T cell crosstalk. Nat Immunol.

[11] Coskun M, Bjerrum JT, Seidelin JB, Nielsen OH (2012). MicroRNAs in inflammatory bowel disease--pathogenesis, diagnostics and therapeutics. World J Gastroenterol.

[12] Tao X, MicroRNA Transcriptome XZ (2013). In swine small intestine during weaning stress. PLoS One.

[13] Mariani V, Palermo S, Fiorentini S, Lanubile A, Giuffra E (2009). Gene expression study of two widely used pig intestinal epithelial cell lines: IPEC-J2 and IPI-2I. Vet Immunol Immunopathol.

[14] Liu D, Yu J, Chen H, Reichman R, Wu H, Jin X (2006). Statistical determination of threshold for cellular division in the CFSE-labeling assay. J Immunol Methods.

[15] Jiang LH, Yang NY, Yuan XL, Zou YJ, Zhao FM, Chen JP (2014). Daucosterol promotes the proliferation of neural stem cells. J Steroid Biochem Mol Biol.

[16] Lian C, Sun B, Niu S, Yang R, Liu B, Lu C, Meng J (2012). A comparative profile of the microRNA transcriptome in immature and mature porcine testes using Solexa deep sequencing. FEBS J.

[17] John B, Enright AJ, Aravin A, Tuschl T, Sander C, Marks DS (2004). Human microRNAs targets. PLoS Biol.

[18] Enright AJ, John B, Gaul U, Tuschl T, Sander C, Marks DS (2003). MicroRNA targets in drosophila. Genome Biol.

[19] David JC, Grongnet JF, Lalles JP (2002). Weaning affects the expression of heat shock proteins in different regions of the gastrointestinal tract of piglets. J Nutr.

[20] Glushkova OV, Novoselova TV, Khrenov MO, Parfenyuk SB, Lunin SM, Fesenko EE (2010). Role of heat shock protein hsp90 in formation of protective reactions in acute toxic stress. Biochemistry (Mosc).

[21] Gallo LI, Lagadari M, Piwien-Pilipuk G, Galigniana MD (2011). The 90-kDa heat-shock protein (Hsp90)-binding immunophilin FKBP51 is a mitochondrial protein that translocates to the nucleus to protect cells against oxidative stress. J Biol Chem.

[22] Peterson LW, Artis D (2014). Intestinal epithelial cells: regulators of barrier function and immune homeostasis. Nat Rev Immunol.

[23] Kozomara A, Griffiths-Jones S (2014). miRBase: annotating high confidence microRNAs using deep sequencing data. Nucleic Acids Res.

[24] Kutty RK, Nagineni CN, Samuel W, Vijayasarathy C, Jaworski C, Duncan T (2013). Differential regulation of microRNA-146a and microRNA-146b-5p in human retinal pigment epithelial cells by interleukin-1β, tumor necrosis factor-α, and interferon-γ. Mol Vis.

[25] Shi C, Zhu L, Chen X, Gu N, Chen L, Zhu L (2014). IL-6 and TNF-α induced obesity-related inflammatory response through transcriptional regulation of miR-146b. J Interf Cytokine Res.

[26] Park H, Huang X, Lu C, Cairo MS, Zhou X (2015). miR-146a and miR-146b regulate human dendritic cell apoptosis and cytokine production by targeting of TRAF6 and IRAK1. J Biol Chem.

[27] Bitar A, De R, Melgar S, Aung KM, Rahman A, Qadri F (2017). Induction of immunomodulatory miR-146a and miR-155 in small intestinal epithelium of vibrio cholerae infected patients at acute stage of cholera. PLoS One.

[28] Liao Y, Zhang M, LÖnnerdal B (2013). Growth factor TGF-b induces intestinal epithelial cell (IEC-6) differentiation: miR-146b as a regulatory component in the negative feedback loop. Genes Nutr.

[29] Chen L, Dai YM, Ji CB, Yang L, Shi CM, GF X (2014). miR-146b is a regulator of human visceral preadipocyte proliferation and differentiation and its expression is altered in human obesity. Mol Cell Endocrinol.

[30] Sun SG, Zheng B, Han M, Fang XM, Li HX, Miao SB (2011). miR-146a and Krüppel-like factor 4 form a feedback loop to participate in vascular smooth muscle cell proliferation. EMBO Rep.

[31] Li J, Shan F, Xiong G, Wang JM, Wang WL, Bai Y (2014). Transcriptional regulation of miR-146b by C/EBPβ LAP2 in esophageal cancer cells. Biochem Biophys Res Commun.

[32] Taganov KD, Boldin MP, Chang KJ, Baltimore D (2006). NF-kappaB-dependent induction of microRNA miR-146, an inhibitor targeted to signaling proteins of innate immune responses. Proc Natl Acad Sci U S A.

[33] Jiang W, Liu J, Dai Y, Zhou N, Ji C, Li X (2015). miR-146b attenuates high-fat diet-induced non-alcoholic steatohepatitis in mice. J Gastroenterol Hepatol.

[34] Roos J, Enlund E, Funcke JB (2015). miR-146a-mediated suppression of the inflammatory response in human adipocytes. Sci Rep.

[35] Habibi F, Soufi FG, Ghiasi R, Khamaneh AM, Alipour MR (2016). Alteration in inflammation-related mir-146a expression in NF-κβ signaling pathway in diabetic rat hippocampus. Adv Pharm Bull.

[36] Zhu Y, Xue Z, Di L (2017). Regulation of miR-146a and TRAF6 in the diagnose of lupus nephritis. Med Sci Monit.

[37] Chassin C, Hempel C, Stockinger S, Dupont A, Kübler JF, Wedemeyer J (2012). microRNA-146a-mediated downregulation of IRAK1 protects mouse and human small intestine against ischemia/reperfusion injury. EMBO Molecular Medicine.

[38] Jiang W, Ni Q, Tan L, Kong L, Lu Y, Xu X (2015). The microRNA-146a/b attenuates acute small-for-size liver graft injury in rats. Liver Int.

[39] Curtale G, Mirolo M, Renzi TA, Rossato M, Bazzoni F, Locati M (2013). Negative regulation of toll-like receptor 4 signaling by IL-10-dependent microRNA-146b. Proc Natl Acad Sci U S A.

